# First external quality assurance program for bloodstream Real-Time PCR monitoring of treatment response in clinical trials of Chagas disease

**DOI:** 10.1371/journal.pone.0188550

**Published:** 2017-11-27

**Authors:** Juan C. Ramírez, Rudy Parrado, Elena Sulleiro, Anabelle de la Barra, Marcelo Rodríguez, Sandro Villarroel, Lucía Irazu, Cristina Alonso-Vega, Fabiana Alves, María A. Curto, Lineth García, Lourdes Ortiz, Faustino Torrico, Joaquim Gascón, Laurence Flevaud, Israel Molina, Isabela Ribeiro, Alejandro G. Schijman

**Affiliations:** 1 Instituto de Investigaciones en Ingeniería Genética y Biología Molecular “Dr. Héctor N. Torres” (INGEBI-CONICET), Buenos Aires, Argentina; 2 Instituto de Investigaciones Biomédicas (IIBISMED), Universidad Mayor de San Simón, Cochabamba, Bolivia; 3 Hospital Universitari Vall d'Hebron, Universitat Autònoma de Barcelona, PROSICS Barcelona, Barcelona, Spain; 4 Instituto Nacional de Enfermedades Infecciosas (INEI)-ANLIS "Dr. Carlos G. Malbrán", Buenos Aires, Argentina; 5 Drugs for Neglected Diseases *initiative* (DND*i*), Geneva, Switzerland; 6 Universidad Autónoma Juan Misael Saracho, Tarija, Bolivia; 7 Fundación CEADES, Cochabamba, Bolivia; 8 ISGlobal, Barcelona Centre for International Health Research (CRESIB), Hospital Clínic-Universitat de Barcelona, Barcelona, Spain; 9 Médecins Sans Frontières Operational Center Barcelona-Athens (OCBA), Barcelona, Spain; Janssen Research and Development, UNITED STATES

## Abstract

Real-Time PCR (qPCR) testing is recommended as both a diagnostic and outcome measurement of etiological treatment in clinical practice and clinical trials of Chagas disease (CD), but no external quality assurance (EQA) program provides performance assessment of the assays in use. We implemented an EQA system to evaluate the performance of molecular biology laboratories involved in qPCR based follow-up in clinical trials of CD. An EQA program was devised for three clinical trials of CD: the E1224 (NCT01489228), a pro-drug of ravuconazole; the Sampling Study (NCT01678599), that used benznidazole, both conducted in Bolivia; and the CHAGASAZOL (NCT01162967), that tested posaconazole, conducted in Spain. Four proficiency testing panels containing negative controls and seronegative blood samples spiked with 1, 10 and 100 parasite equivalents (par. eq.)/mL of four *Trypanosoma cruzi* stocks, were sent from the Core Lab in Argentina to the participating laboratories located in Bolivia and Spain. Panels were analyzed simultaneously, blinded to sample allocation, at 4-month intervals. In addition, 302 random blood samples from both trials carried out in Bolivia were sent to Core Lab for retesting analysis. The analysis of proficiency testing panels gave 100% of accordance (within laboratory agreement) and concordance (between laboratory agreement) for all *T*. *cruzi* stocks at 100 par. eq./mL; whereas their values ranged from 71 to 100% and from 62 to 100% at 1 and 10 par. eq./mL, respectively, depending on the *T*. *cruzi* stock. The results obtained after twelve months of preparation confirmed the stability of blood samples in guanidine-EDTA buffer. No significant differences were found between qPCR results from Bolivian laboratory and Core Lab for retested clinical samples. This EQA program for qPCR analysis of CD patient samples may significantly contribute to ensuring the quality of laboratory data generated in clinical trials and molecular diagnostics laboratories of CD.

## Introduction

Chagas disease (CD), caused by the kinetoplastid flagellate *Trypanosoma cruzi*, has been considered to be “the most neglected of the neglected diseases” given the research and development gaps related to diagnosis and treatment [1]. Accurate diagnostics tools, as well as surrogate markers of parasitological response to treatment, are priorities in CD research and development [1–3].

In order to develop a reliable laboratory tool for diagnosis and treatment follow-up, several difficulties need to be addressed, such as the low and intermittent number of circulating parasites during the chronic phase of infection as well as parasite genotype diversity, since six Discrete Typing Units (DTUs), TcI-TcVI, are unevenly distributed in different endemic regions [4,5]. Quantitative Real-Time PCR (qPCR) based assays were developed aiming to fill these gaps, but their application in clinical practice required prior analytical and clinical validation studies that have been recently accomplished [6,7].

The most widely applied qPCR standard operating procedure (SOP) for detection and quantification of *T*. *cruzi* DNA includes DNA extraction from 300 μL of guanidine-EDTA-blood samples using glass-fiber commercial columns. This is followed by duplex qPCR using TaqMan probes targeted to *T*. *cruzi* Satellite DNA (SatDNA) and an internal amplification control (IAC) [6,7]. This method has been used to follow-up parasite response to treatment with different compounds and regimens, such as benznidazole [8] and E1224, a water-soluble ravuconazole pro-drug [9].

External quality assurance (EQA) programs designed to provide performance assessment for molecular diagnostics assays have been carried out for several viral [10–21], bacterial [22,23] and parasite pathogens [24–26]. With this aim in mind, a multicenter study was carried out to analyze the performance, of existing PCR methods for *T*. *cruzi* DNA detection in 2008 [27]. However, there are still no formal EQA programs for qPCR performance assessment of laboratories involved in molecular diagnosis or clinical trials of CD in which qPCR assay is considered as a primary endpoint.

This work aimed to evaluate the performance of SatDNA qPCR methods used as primary endpoints during three clinical trials of chronic CD patients conducted in Bolivia and Spain, through an EQA program specially devised for this purpose. The EQA program included (a) panels of non-infected blood samples spiked with known quantities of *T*. *cruzi* epimastigotes belonging to stocks representative of different DTUs and (b) peripheral blood samples from CD patients enrolled in the two trials conducted in Bolivia that were retested by a reference laboratory to address inter-laboratory concordance of qPCR findings.

To our knowledge, this is the first implementation of an EQA program for monitoring the use of *T*. *cruzi* based qPCR as a surrogate biomarker of treatment response in clinical trials of CD.

## Materials and methods

### Ethics statement

The clinical trials including the sampling requirements were approved by the Ethical Review Boards of Universidad Mayor de San Simón, Fundación CEADES, Hospital Clínic, Médecins Sans Frontières and Hospital Universitari Vall d'Hebron; following the principles expressed in the Declaration of Helsinki. Written informed consent forms were signed by the study volunteers (no minor subjects were included in these trials). All samples were anonymized before being processed.

### Clinical trials

**E1224 (NCT01489228):** This trial was designed by Drugs for Neglected Diseases *initiative* (DND*i*). A phase II proof-of-concept double-blinded randomized trial aimed to assess the safety and efficacy of three oral regimens of E1224, a pro-drug of ravuconazole, compared to benznidazole (BZN) and placebo, during 60 days of treatment. Chronic CD patients (N = 231) from the cities of Cochabamba and Tarija, both in Bolivia, were part of this trial.

**Sampling Study (NCT01678599):** This trial launched by DND*i* and Médecins Sans Frontières aimed to evaluate sampling conditions for qPCR monitoring of chronic CD patients (N = 205) treated with BZN during 60 days. This study was carried out in the locality of Aiquile, Bolivia.

**CHAGASAZOL (NCT01162967):** This trial was performed by the Hospital Universitari Vall d'Hebron in Barcelona, Spain. A phase II randomized open trial aimed to assess the efficacy, safety and side-effect profile of two oral regimens of posaconazole, compared to BZN, during 60 days of treatment. Seventy-five of the 78 chronic CD patients enrolled in this trial were from Bolivia.

### External quality assurance program design

#### Proficiency testing panels (PTPs)

The design of the PTPs took into account those parasite stocks that were previously used during SatDNA qPCR validation [6], and TcI and TcV stocks prevailing in the geographical region of the enrolled patients [6]. Accordingly, TcII, TcIII and TcIV stocks were not included.

Four blinded panels containing seronegative human blood samples spiked with 1, 10 and 100 parasite equivalents (par. eq.)/mL of cultured epimastigotes from K98 (TcIa), Sylvio X10 Cl1 (TcId), LL014-1-R1 Cl1 (TcV) and CL-Brener (TcVI) *T*. *cruzi* stocks, and four negative controls were prepared by the Laboratory of Molecular Biology of Chagas Disease at INGEBI-CONICET, Buenos Aires, Argentina (Core Lab).

After being spiked, blood samples were immediately mixed with an equal volume of Guanidine Hydrochloride 6M-EDTA 0.2M pH 8.0 buffer and incubated for 48 hours at room temperature. Finally, guanidine-EDTA-blood (GEB) samples were aliquoted and stored at 4°C until DNA extraction and qPCR analysis. Each PTP was analyzed at the same time, blinded to sample allocation, at 4-month intervals (0, 4, 8 and 12 months), by Core Lab and the laboratories involved in the clinical trials from Bolivia, with two operators (LabB-Op1 and LabB-Op2), and Spain (LabC).

#### Retesting samples analysis

Only LabB provided clinical samples for retesting analysis. For this purpose, 302 GEB samples were selected at random and aliquots of 1 mL were sent to Core Lab. There were 173 samples from the E1224 trial (92 from Cochabamba and 81 from Tarija) and 129 samples from the Sampling Study trial.

### DNA extraction procedures

**LabB and Core Lab:** 300 μL GEB samples were processed using the High Pure PCR Template Preparation kit (Roche Diagnostics, Indianapolis, IN) and eluted in 100 μL elution buffer as described in Duffy et al. 2013 [6].

**LabC:** 200 μL GEB samples were processed using the NucliSENS easyMAG system (bioMérieux, Marcy l'Etoile, France) and eluted in 50 μL elution buffer.

In all cases, the eluted DNA was stored at -20°C until use in qPCR analysis.

### Satellite DNA Real-Time PCR procedures

**LabB and Core Lab:** a duplex qPCR targeted to *T*. *cruzi* SatDNA and IAC sequence, previously described in Duffy et al. 2013 [6], was used. The qPCR reactions were carried out using the FastStart Universal Probe Master Mix (Roche Diagnostics, Mannheim, Germany) with 5 μL eluted DNA in 20 μL final volume. The qPCRs were performed in triplicate, except for PTP1 samples analyzed at Core Lab, which were performed in duplicate.

Clinical samples from the retesting study were analyzed in duplicate and, in case both replicates gave non-detectable results, a third qPCR assay was run according to clinical trial protocols. For both laboratories, standard curves were plotted with 1/10 serial dilutions of total DNA obtained from the same stock of GEB seronegative sample spiked with 10^5^ par. eq./mL of TcV cultured epimastigotes. LabB and Core Lab qPCR methods have a Limit of Detection of 0.20 and 0.70 par. eq./mL, respectively.

Cycling conditions were a first step of 10 minutes at 95°C followed by 40 cycles at 95°C for 15 seconds and 58°C for 1 minute. Amplifications were carried out in a Rotor-Gene Q (Corbett Life Science, Cambridgeshire, United Kingdom) and an ABI7500 (Applied Biosystems, Foster City, CA) Real-Time PCR devices, at LabB and Core Lab, respectively.

**LabC-SOP1:** a duplex qPCR targeted to *T*. *cruzi* SatDNA and human RNase P gene, previously described in Piron et al. 2007 [28], was used. The qPCR reactions were carried out in duplicate using the TaqMan Universal PCR Master Mix and 1x TaqMan RNase P Control Reagents kit (Applied Biosystems), with 5 μL eluted DNA in 20 μL final volume.

Cycling conditions were a first step of 2 minutes at 50°C, a second step of 10 minutes at 95°C followed by 40 cycles at 95°C for 15 seconds and 58°C for 1 minute. Amplifications were carried out in a SmartCycler Real-Time PCR system (Cepheid, Sunnyvale, CA).

**LabC-SOP2:** used a modification of the LabC-SOP1 method, implemented only during PTP4 analysis as follows: final concentrations in the PCR mixture were 400 nM cruzi1 and cruzi2 SatDNA primers, and 100 nM cruzi3 TaqMan probe. The qPCR reactions were carried out in duplicate using the QuantiTect Multiplex PCR kit (Qiagen, Manchester, United Kingdom), with 5 μL eluted DNA in 25 μL final volume.

Cycling conditions were a first step of 15 minutes at 95°C followed by 40 cycles at 95°C for 15 seconds and 58°C for 1 minute. Amplifications were carried out in a CFX Real-Time PCR detection system (Bio-Rad, Hercules, CA).

### PCR quality controls

A negative control and two positive controls containing 10 and 1 fg/μL *T*. *cruzi* CL-Brener DNA were included in every run, as recommended [29].

### Internal amplification controls

A pZErO-2 recombinant plasmid containing an inserted sequence of *Arabidopsis thaliana* aquaporin was used as an exogenous amplification control by LabB and Core Lab [30]; whereas the human RNase P gene was used as an endogenous control by LabC [28].

### Statistical analysis

Accordance (within laboratory agreement) and concordance (between laboratory agreement) were calculated for qualitative analysis of proficiency testing results [31]. Accordance and concordance are defined as the percentage chance that two identical test materials analyzed by the same laboratory or sent to different laboratories will both be given the same result (i.e. qPCR positive or negative), respectively. In addition, the concordance odds ratio (COR) was calculated as follows [COR = accordance x (100—concordance)/ concordance x (100—accordance)] to assess the degree of between laboratory variation [31]. Standard errors and 95% confidence intervals of accordance, concordance and COR were estimated by bootstrap analysis (500 replicates); all parameters were calculated using an Excel application available by e-mail from Langton et al. [31]. Moreover, McNemar's test was used to compare the qualitative results per sample among all laboratories, considering as positive those samples with at least one positive qPCR replicate. Kruskal-Wallis non-parametric analysis of variance was used to compare the medians of SatDNA Ct (threshold cycle) values of detectable samples, grouped by laboratory, *T*. *cruzi* stock and number of proficiency testing panel, using SPSS Statistics for Windows V17.0 (SPSS, Chicago, IL).

On the other hand, Cohen *k* coefficient [32] and McNemar's test were used to analyze the closeness of the agreement and the differences between LabB and Core Lab qualitative results for the retested clinical samples, respectively. Furthermore, Bland-Altman bias plot [29] and paired *t* test were used to analyze the closeness of the agreement between the quantitative results and the means of parasitic loads of quantifiable samples from both laboratories, respectively. Finally, Tukey's criterion was used to detect samples with outlier Ct values of IAC (Cts> 75th percentile + 1.5 x interquartile distance of median Ct) at LabB and Core Lab [33].

## Results

### Analysis of proficiency testing panels

This study included four panels of negative controls and samples spiked with four *T*. *cruzi* stocks belonging to three different DTUs (TcI, TcV and TcVI) and two different TcI mini-exon-based genotypes (TcIa and TcId), at three different concentrations. The accordance and concordance analysis of the qualitative SatDNA qPCR results obtained by each participating laboratory for the spiked samples are shown in Table 1.

**Table 1:** Accordance and concordance analysis of SatDNA qPCR qualitative results for proficiency testing panels.

*T*. *cruzi*	Laboratory	Total of	Number of Positive Replicates		
stock		Replicates	1 par. eq./mL	10 par. eq./mL	100 par. eq./mL
TcIa K98	Core Lab	11	11	11	11
	LabB-Op1	12	12	12	12
	LabB-Op2	12	12	12	12
	LabC-SOP1	8	8	8	8
	Accordance [95CI] (%)		100 [100–100]	100 [100–100]	100 [100–100]
	Concordance [95CI] (%)		100 [100–100]	100 [100–100]	100 [100–100]
	COR [95CI]		1 [1–1]	1 [1–1]	1 [1–1]
TcId	Core Lab	11	1	10	11
Sylvio	LabB-Op1	12	2	8	12
X10 Cl1	LabB-Op2	12	1	11	12
	LabC-SOP1	8	1	4	8
	Accordance [95CI] (%)		77.67 [64.19–94.66]	67.91 [58.83–84.19]	100 [100–100]
	Concordance [95CI] (%)		79.36 [64.60–93.19]	62.06 [49.56–77.91]	100 [100–100]
	COR [95CI]		0.90 [0.84–1.56]	1.29 [0.97–3.04]	1 [1–1]
TcV	Core Lab	11	1	11	11
LL014-1-	LabB-Op1	12	2	10	12
R1 Cl1	LabB-Op2	12	4	10	12
	LabC-SOP1	8	0	6	8
	Accordance [95CI] (%)		71.16 [62.33–86.29]	75.81 [65.58–91.63]	100 [100–100]
	Concordance [95CI] (%)		72.38 [58.64–87.06]	75.29 [59.74–90.99]	100 [100–100]
	COR [95CI]		0.94 [0.85–1.62]	1.03 [0.88–2.14]	1 [1–1]
TcVI CL-	Core Lab	11	9	11	11
Brener	LabB-Op1	12	10	12	12
	LabB-Op2	12	12	12	12
	LabC-SOP1	8	6	8	8
	Accordance [95CI] (%)		76.74 [68.13–91.63]	100 [100–100]	100 [100–100]
	Concordance [95CI] (%)		75.00 [60.96–90.70]	100 [100–100]	100 [100–100]
	COR [95CI]		1.10 [0.96–2.00]	1 [1–1]	1 [1–1]

par. eq./mL: parasite equivalents in 1 mL of blood; 95CI: 95% confidence interval; COR: Concordance Odds Ratio

In general, LabB-Op2 had higher positivity [122 out of 144 replicates (84.72%)] than Core Lab [109 out of 132 replicates (82.58%)], LabB-Op1 [116 out of 144 replicates (80.56%)], and LabC-SOP1 [73 out of 96 replicates (76.04%)]. Grouping the results for all *T*. *cruzi* stocks gave 100% samples showing within and between laboratory agreement at 100 par. eq./mL, this decreased to 84.15 and 49.65% at 10 par. eq./mL, and to 82.65 and 50.05% at 1 par. eq./mL, respectively.

On the other hand, the comparison of qualitative SatDNA qPCR results per sample, taking into account that a clinical sample was considered positive if at least one qPCR replicate had a positive result, did not show significant differences among all laboratories (p> 0.05). In addition, all of the 16 negative samples included in the PTPs gave non-detectable results for all qPCR replicates for each participating laboratory (100% concordance and specificity) (S1 Dataset).

#### Analysis by *T*. *cruzi* stock

The results obtained varied depending on the *T*. *cruzi* stock. 100% accordance and concordance was obtained for the three concentrations of TcIa stock, and at 10 and 100 par. eq./mL of TcVI stock. In contrast, for TcVI stock, the concentration of 1 par. eq./mL gave an accordance of 76.74% and a concordance of 75.00%. On the other hand, analysis of the TcId and TcV samples gave lower accordance and concordance values: 77.67% and 71.16%, and 79.36% and 72.38% at 1 par. eq./mL, and 67.91% and 75.81%, and 62.06% and 75.29% at 10 par. eq./mL, respectively; except at 100 par. eq./mL of both *T*. *cruzi* stocks, which showed 100% accordance and concordance (Table 1). Finally, the grade of agreement was higher between laboratories than within them, at 1 par. eq./mL (COR< 1), and the opposite was the case at 10 par. eq./mL (COR> 1) for TcId and TcV samples; whereas at 1 par. eq./mL of TcVI stock, accordance was higher than concordance (COR> 1).

**Fig 1:**
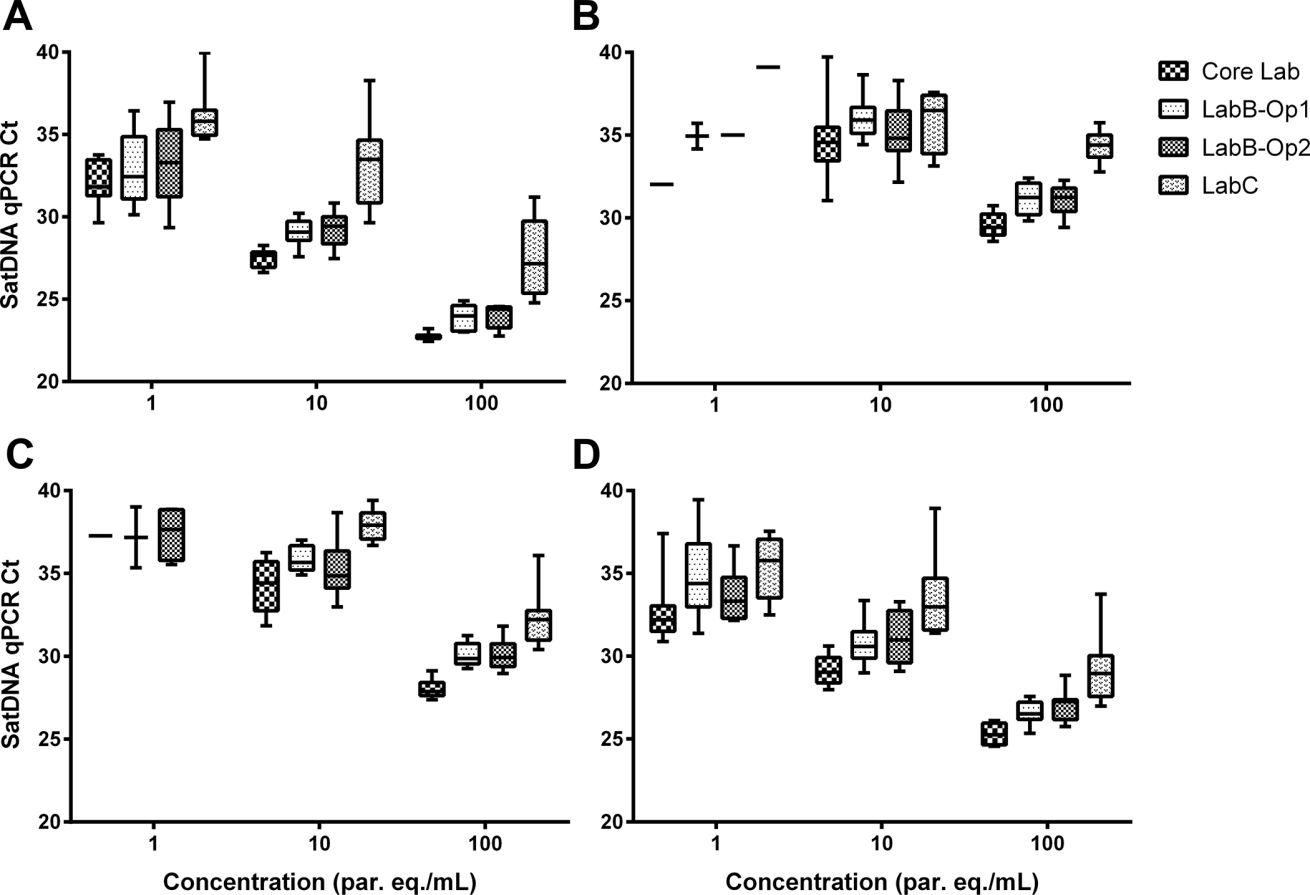
Comparison of intra- and inter-laboratory SatDNA qPCR results for proficiency testing panels analysis based on *T*. *cruzi* stocks. A: K98 (TcIa); B: Sylvio X10 Cl1 (TcId); C: LL014-1-R1 Cl1 (TcV); D: CL-Brener (TcVI); Ct: Cycle threshold; SatDNA qPCR: Satellite DNA Real-Time PCR; par. eq./mL: parasite equivalents in 1 mL of blood.

Fig 1 presents the comparative analysis of SatDNA qPCR Ct values for each *T*. *cruzi* stock. As expected, there were higher Ct values and data dispersion at lower parasite concentrations, but also for TcId and TcV stocks compared to TcIa and TcVI stocks. Moreover, for all *T*. *cruzi* stocks and concentrations, higher Ct values and data dispersion were observed for LabC than for LabB and for LabB compared to Core Lab.

Statistical analysis showed that there were significant differences within each laboratory for all *T*. *cruzi* stocks at 10 and 100 par. eq./mL (p< 0.05). However, no difference was found between TcId and TcV stocks at 10 par. eq./mL at all laboratories, as well as between TcIa and TcId, TcIa and TcVI, and TcId and TcVI stocks at 10 par. eq./mL, and TcIa and TcVI stocks at 100 par. eq./mL at LabC (p> 0.05). Furthermore, there were significant inter-laboratorial differences for each *T*. *cruzi* stock at 10 and 100 par. eq./mL (p< 0.05); except for TcId and TcV stocks at 10 par. eq./mL among all laboratories and between LabB-Op2 and Core Lab, respectively (p> 0.05). There were no significant differences between both operators at LabB for all *T*. *cruzi* stocks and concentrations (p> 0.05).

#### Analysis by number of proficiency testing panel

**Fig 2:**
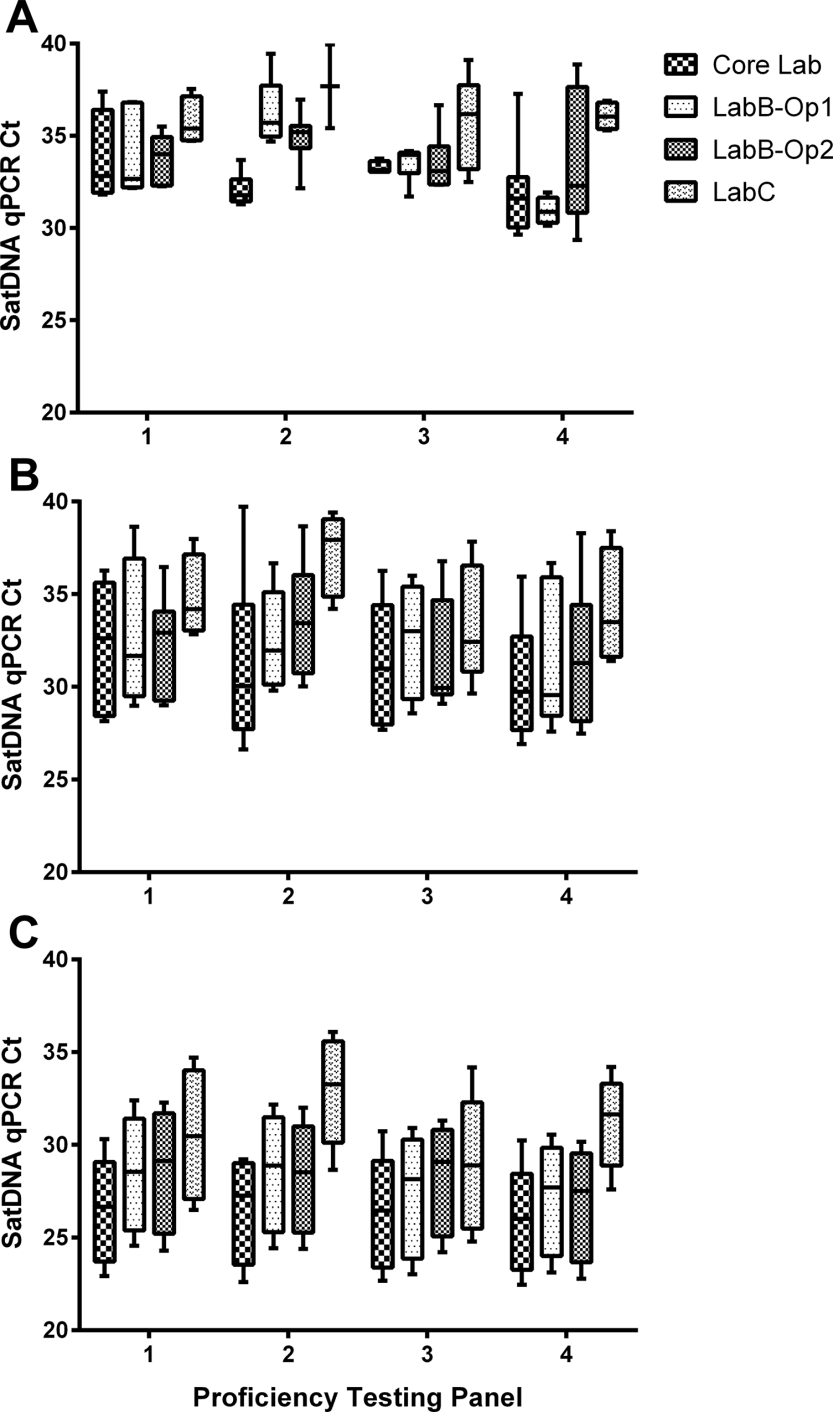
Comparison of intra- and inter-laboratory SatDNA qPCR results for proficiency testing panels analysis based on number of panel. A: 1 par. eq./mL; B: 10 par. eq./mL; C: 100 par. eq./mL; Ct: Cycle threshold; SatDNA qPCR: Satellite DNA Real-Time PCR; par. eq./mL: parasite equivalents in 1 mL of blood.

Fig 2 shows the comparative analysis of SatDNA qPCR Ct values obtained for each PTP. In this case, as for the previous analysis, at the 1 par. eq./mL concentration there were higher differences among all panels at each laboratory than at other concentrations. However, at 10 and 100 par. eq./mL the Ct values and data dispersion of each PTP were very sustained during the test period (twelve months) at all laboratories; except at LabC, which showed small variations from one panel to another. Contrary to expectation, PTP4 showed the lowest Ct values for all parasite concentrations at all laboratories; except at LabC.

In consequence, statistical analysis only found within-laboratory significant differences for LabB-Op1 among all panels at 1 par. eq./mL concentration (p< 0.05); except between PTP1 and PTP3 (p> 0.05). In the same way, inter-laboratory significant differences were only detected for PTP2 between LabC and Core Lab for all concentrations, and between LabB-Op1 and Core Lab at 1 par. eq./mL; as well as for PTP4 between LabC and Core Lab, and between LabB-Op2 and LabC at 100 par. eq./mL (p< 0.05).

Before analysis of PTP4, LabC modified its qPCR SOP to improve the sensitivity of its method. This optimization included an adjustment of primers and probe concentrations, and testing different PCR mixesto reach the final procedure (LabC-SOP2). Table 2 compares the qPCR results obtained by LabC using the modified SOP with those from LabC (original SOP) and Core Lab for PTP4. In general, the modified SOP allowed LabC to reduce their SatDNA Ct values to around 4 units, getting closer to Core Lab Cts. Moreover, with the use of the new SOP, LabC was able to detect three additional positive samples (1 and 10 par. eq./mL for TcId stock, and 1 par. eq./mL for TcV stock) that had given non-detectable results using the original SOP.

**Table 2:** Comparison of SatDNA qPCR LabC-SOP1 and -SOP2 results for proficiency testing panel 4 with those obtained at Core Lab.

*T*. *cruzi*	Concentration	Core Lab			LabC-SOP1		LabC-SOP2	
stock	(par. eq./mL)	Ct1	Ct2	Ct3	Ct1	Ct2	Ct1	Ct2
TcIa K98	0	ND	ND	ND	ND	ND	ND	ND
	1	31.79	29.75	29.64	35.30	36.50	30.85	31.76
	10	26.94	26.91	27.55	31.70	33.40	27.64	27.91
	100	22.45	22.83	22.78	31.20	27.60	23.06	22.83
TcId	0	ND	ND	ND	ND	ND	ND	ND
Sylvio	1	ND	ND	32.03	ND	ND	ND	37.39
X10 Cl1	10	32.62	35.21	31.05	ND	ND	37.26	34.35
	100	28.96	30.23	28.58	33.50	34.20	29.23	30.20
TcV	0	ND	ND	ND	ND	ND	ND	ND
LL014-1-	1	ND	37.28	ND	ND	ND	34.55	ND
R1 Cl1	10	32.75	35.95	31.85	37.20	38.40	34.21	34.07
	100	27.38	27.60	28.04	32.70	32.10	28.99	28.17
TcVI CL-	0	ND	ND	ND	ND	ND	ND	ND
Brener	1	33.01	30.88	31.41	35.60	36.90	33.03	32.31
	10	28.41	28.39	27.98	33.60	31.40	28.65	29.14
	100	24.63	24.65	24.57	29.10	28.80	25.49	25.57

par. eq./mL: parasite equivalents in 1 mL of blood; SOP: Standard Operating Procedure; Ct: Cycle threshold; ND: non-detectable

### Analysis of retesting samples

As part of this study, 302 GEB samples, 173 from E1224 and 129 from Sampling Study trials, were randomly selected and sent from LabB to Core Lab to be retested. One hundred and thirty-seven (45.36%) and 129 (42.72%) out of 302 samples had detectable qPCR results for LabB and Core Lab, respectively; 112 of these samples were detectable by both laboratories, whereas 25 and 17 samples were detectable (but non-quantifiable) only by LabB or Core Lab, respectively (p> 0.05). Moreover, the estimation of *k* coefficient gave 0.72 (95%CI, 0.64–0.80), indicating that the strength of agreement between the qualitative results obtained by both laboratories was good.

**Fig 3:**
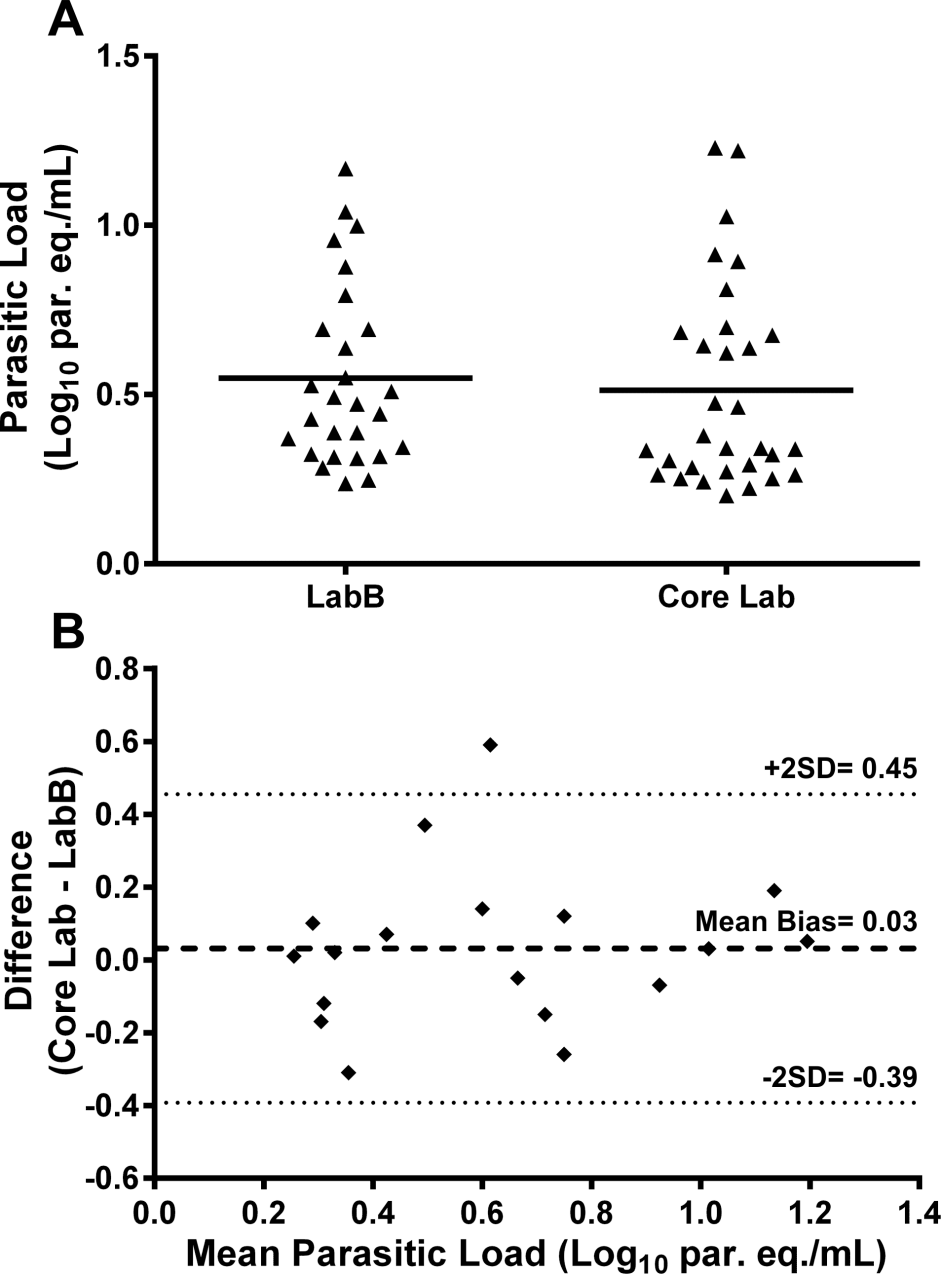
Comparison of quantifiable SatDNA qPCR results for retesting samples analysis. **Parasitic loads obtained by LabB and Core Lab (A) and Bland-Altman bias plot as a measure of the degree of agreement between the results of both laboratories (B).** par. eq./mL: parasite equivalents in 1 mL of blood; SatDNA qPCR: Satellite DNA Real-Time PCR; SD: standard deviation.

Fig 3A shows the parasitic loads of 27 out of 135 (20.00%) and 31 out of 127 (24.41%) qPCR quantifiable samples determined by LabB and Core Lab, respectively.

The degree of agreement between the parasitic loads of the 18 samples with quantifiable results at both laboratories is represented as a Bland-Altman bias plot in Fig 3B. As shown, the mean bias was determined to be 0.03 Log_10_ par. eq./mL, indicating a systematic bias of 1.07-fold par. eq./mL between both laboratories. This difference was not considered to be statistically significant since the 95% confidence interval [0.45-(-0.39) Log_10_ par. eq./mL], expressed as mean bias ± 2 standard deviation, contains zero (no difference). Furthermore, no significant differences were found between the parasitic loads of LabB (3.30 [2.17–7.20] par. eq./mL) and Core Lab (4.38 [2.17–7.48] par. eq./mL) using paired *t* test analysis (p> 0.05).

## Discussion

Accurate diagnostics tools for detection of *T*. *cruzi* infection and surrogate markers of parasitological response to treatment are priorities in CD research and development [1–3]. To reach these goals, several difficulties need to be addressed, such as the low and intermittent number of circulating parasites during the chronic phase of infection and *T*. *cruzi* genotype diversity, since six DTUs are unevenly distributed in different endemic regions [4,5]. qPCR-based assays for *T*. *cruzi* DNA detection and quantification in CD patients have been developed to fill these gaps, but their application in clinical practice has required analytical and clinical validation studies [6,7].

EQA programs offer a laboratory measurement tool for ensuring accurate, timely, clinically appropriate and useful information. They also provide sponsoring and regulatory agencies with confidence that laboratory data are generated with a rigor that will support product licensure and ensure that samples from clinical trials will be analyzed in a system that guarantees trial volunteer safety [34]. Moreover, participation in EQA programs makes data and information available which allows for the comparison of performance and results among all participating laboratories, provides early warning for systematic problems related with reagents or procedures, affords objective evidence of testing quality, identifies training needs, and detects areas that need improvement [35].

Several EQA programs have been carried out for viral, bacterial and parasite pathogens such as Cytomegalovirus [10], HIV-1 [11,12,19], JC virus [13], Hepatitis C [14], G [17] and B [18] viruses, Enterovirus [15], Herpes Simplex virus [16], Dengue virus [20], Yellow Fever virus [21], *Mycobacterium tuberculosis* [22], *Bordetella pertussis* [23], Toxoplasmosis [24], Leishmaniasis [25], and Plasmodium [26].

The first initiative made in this direction in CD was an international collaborative study launched by 26 expert laboratories from 16 countries. It evaluated the ability of their existing PCR procedures to detect *T*. *cruzi* DNA from a blind panel containing genomic DNA from several strains and spiked blood samples with different parasite concentrations, as well as blood samples from seropositive patients and non-infected controls [27]. Later on, an international workshop validated two duplex qPCR strategies using TaqMan probes for detection and quantification of bloodstream parasite loads in samples from CD patients [7], that had been used to assess parasitic response to treatment in recent clinical trials [8,9]. In this context, we have designed and implemented for the first time an EQA program that included proficiency testing and retesting analyses for *T*. *cruzi* qPCR performance assessment in three clinical trials of CD.

The design of the PTPs took into account those parasite stocks that were previously used during SatDNA qPCR validation [6]: CL-Brener, reference stock for TcVI, and Sylvio X10, reference stock for TcI [4]. In addition, we aimed to include stocks belonging to DTUs circulating in those regions where the patients enrolled in these clinical trials became infected. Consequently, we selected a parasite stock belonging to TcV, the predominant DTU in Bolivia, and another strain belonging to TcI, which is also common in Bolivia [5,36]. These strains harbor different copy numbers of the repetitive satellite DNA sequence employed as a molecular target, and this diversity can explain the variability in qPCR positivity obtained in PTPs analysis. This feature was also reflected in the intra- and inter-laboratory variability observed among stocks, particularly for those samples with lower parasite concentrations, and in the dispersion of Ct values when all stocks were compared together.

Other factors, such as differences between the DNA extraction and qPCR methods used by LabC compared to those used in the other two laboratories, as well as the different thermocyclers used, may have added more variability to the results. Nevertheless, it is worth mentioning that the qualitative qPCR results per sample did not show significant differences among all participant laboratories.

This study also demonstrated the stability of blood samples treated with guanidine-EDTA buffer for 48 hours and conserved at 4°C for 12 months. No intra-laboratory differences were observed and despite overseas transportation of PTPs, low variability was detected in inter-laboratory comparisons. Moreover, this study was useful in improving molecular diagnostics laboratory practices and tuning the methodology, in particular in the case of LabC, which improved the sensitivity of its qPCR assay on the basis of their results during PTPs analysis.

Finally, the retesting study did not show significant differences in terms of qPCR positivity and parasitic loads between LabB and Core Lab, as shown by McNemar's and paired *t* test analyses, respectively. In sum, overall accordance and concordance of outcomes reported by the participant laboratories demonstrate the reliability of the SatDNA qPCR methods used and encourage expanding their use in future clinical trials of CD.

Although molecular-based techniques have proven useful in research and clinical laboratories, as well as in clinical trial settings, it is hard to envision their application in public health care areas in the absence of appropriate EQA programs. We believe that this work represents a significant contribution towards the achievement of this requisite.

## Supporting information

S1 Dataset Proficiency testing panels results database. (RAR)

## References

[1] TDR/WHO. Research Priorities for Chagas Disease, Human African Trypanosomiasis and Leishmaniasis. WHO Technical Report Series. 2012; 975. 23484340

[2] Pinazo M-J, Thomas MC, Bua J, Perrone A, Schijman A-G, Viotti R-J, et al Biological markers for evaluating therapeutic efficacy in Chagas disease, a systematic review. Expert Rev Anti Infect Ther. 2014; 12: 479–496. doi: 10.1586/14787210.2014.899150 24621252 10.1586/14787210.2014.899150

[3] Porras AI, Yadon ZE, Altcheh J, Britto C, Chaves GC, Flevaud L, et al Target Product Profile (TPP) for Chagas Disease Point-of-Care Diagnosis and Assessment of Response to Treatment. PLoS Negl Trop Dis. 2015; 9: e0003697 doi: 10.1371/journal.pntd.0003697 26042730 10.1371/journal.pntd.0003697 PMC4456144

[4] Zingales B, Andrade SG, Briones MRS, Campbell DA, Chiari E, Fernandes O, et al A new consensus for *Trypanosoma cruzi* intraspecific nomenclature: second revision meeting recommends TcI to TcVI. Mem Inst Oswaldo Cruz. 2009; 104: 1051–4. 20027478 10.1590/s0074-02762009000700021

[5] Zingales B, Miles MA, Campbell DA, Tibayrenc M, Macedo AM, Teixeira MMG, et al The revised *Trypanosoma cruzi* subspecific nomenclature: rationale, epidemiological relevance and research applications. Infect Genet Evol. 2012; 12: 240–53. doi: 10.1016/j.meegid.2011.12.009 22226704 10.1016/j.meegid.2011.12.009

[6] Duffy T, Cura CI, Ramirez JC, Abate T, Cayo NM, Parrado R, et al Analytical performance of a multiplex Real-Time PCR assay using TaqMan probes for quantification of *Trypanosoma cruzi* satellite DNA in blood samples. PLoS Negl Trop Dis. 2013; 7: e2000 doi: 10.1371/journal.pntd.0002000 23350002 10.1371/journal.pntd.0002000 PMC3547845

[7] Ramírez JC, Cura CI, Moreira C, Lages-Silva E, Juiz N, Velázquez E, et al Analytical Validation of Quantitative Real-Time PCR Methods for Quantifi cation of *Trypanosoma cruzi* DNA in Blood Samples from Chagas Disease Patients. J Mol Diagn. 2015; 17: 605–15. doi: 10.1016/j.jmoldx.2015.04.010 26320872 10.1016/j.jmoldx.2015.04.010 PMC4698797

[8] Alvarez MG, Hernandez Y, Bertocchi G, Fernandez M, Lococo B, Ramirez JC, et al New Scheme of Intermittent Benznidazole Administration in Patients Chronically Infected with *Trypanosoma cruzi*: a Pilot Short-Term Follow-Up Study with Adult Patients. Antimicrob Agents Chemother. 2015; 60: 833–837. doi: 10.1128/AAC.00745-15 26596935 10.1128/AAC.00745-15 PMC4750658

[9] Torrico F, Gascon J, Ortiz L, Alonso-Vega C, Pinazo M, Schijman A, et al Treatment of adult chronic indeterminate Chagas disease: proof-of-concept randomized placebo-controlled study of benznidazole and three E1224 dosing regimens. Lancet Infect Dis. 2017; Forthcoming. 10.1016/S1473-3099(17)30538-8 PMC7612561 29352704

[10] Grundy JE, Ehrnst A, Einsele H, Emery VC, Hebart H, Prentice HG, et al A three-center European external quality control study of PCR for detection of cytomegalovirus DNA in blood. J Clin Microbiol. 1996; 34: 1166–1170. 8727896 10.1128/jcm.34.5.1166-1170.1996 PMC228975

[11] Yen-Lieberman B, Brambilla D, Jackson B, Bremer J, Coombs R, Cronin M, et al Evaluation of a quality assurance program for quantitation of human immunodeficiency virus type 1 RNA in plasma by the AIDS Clinical Trials Group virology laboratories. J Clin Microbiol. 1996; 34: 2695–2701. 8897167 10.1128/jcm.34.11.2695-2701.1996 PMC229388

[12] Schweiger B, Pauli G, Zeichhardt H, Kucherer C. A multicentre quality assessment study to monitor the performance of HIV-1 PCR. J Virol Methods. 1997; 67: 45–55. 9274817 10.1016/s0166-0934(97)00075-x

[13] Weber T, Klapper PE, Cleator GM, Bodemer M, Luke W, Knowles W, et al Polymerase chain reaction for detection of JC virus DNA in cerebrospinal fluid: a quality control study. European Union Concerted Action on Viral Meningitis and Encephalitis. J Virol Methods. 1997; 69: 231–237. 9504768 10.1016/s0166-0934(97)00152-3

[14] Robertson JS. International standardization of gene amplification technology. Biologicals. 1998; 26: 111–113. doi: 10.1006/biol.1998.0136 9811515 10.1006/biol.1998.0136

[15] van Loon AM, Cleator GC, Ras A. External quality assessment of enterovirus detection and typing. European Union Concerted Action on Virus Meningitis and Encephalitis. Bull World Health Organ. 1999; 77: 217–223. 10212511 PMC2557629

[16] Hirsch HH, Bossart W. Two-centre study comparing DNA preparation and PCR amplification protocols for herpes simplex virus detection in cerebrospinal fluids of patients with suspected herpes simplex encephalitis. J Med Virol. 1999; 57: 31–35. 9890419 10.1002/(sici)1096-9071(199901)57:1<31::aid-jmv5>3.0.co;2-j

[17] Lefrere JJ, Lerable J, Mariotti M, Bogard M, Thibault V, Frangeul L, et al Lessons from a multicentre study of the detectability of viral genomes based on a two-round quality control of GB virus C (GBV-C)/hepatitis G virus (HGV) polymerase chain reaction assay. J Virol Methods. 2000; 85: 117–124. 10716345 10.1016/s0166-0934(99)00160-3

[18] Valentine-Thon E, van Loon AM, Schirm J, Reid J, Klapper PE, Cleator GM. European proficiency testing program for molecular detection and quantitation of hepatitis B virus DNA. J Clin Microbiol. 2001;39: 4407–4412. doi: 10.1128/JCM.39.12.4407-4412.2001 11724853 10.1128/JCM.39.12.4407-4412.2001 PMC88557

[19] Nowicki MJ, Benning L, Bremer JW, Meyer WA 3rd, Hanson C, Brambilla D, et al Longitudinal variability of human immunodeficiency virus type 1 RNA viral load measurements by nucleic acid sequence-based amplification and NucliSens assays in a large multicenter study. J Clin Microbiol. 2001; 39: 3760–3763. doi: 10.1128/JCM.39.10.3760-3763.2001 11574612 10.1128/JCM.39.10.3760-3763.2001 PMC88428

[20] Domingo C, Niedrig M, Teichmann A, Kaiser M, Rumer L, Jarman RG, et al 2nd International external quality control assessment for the molecular diagnosis of dengue infections. PLoS Negl Trop Dis. 2010; 4: e833 doi: 10.1371/journal.pntd.0000833 20957194 10.1371/journal.pntd.0000833 PMC2950135

[21] Domingo C, Escadafal C, Rumer L, Mendez JA, Garcia P, Sall AA, et al First international external quality assessment study on molecular and serological methods for yellow fever diagnosis. PLoS One. 2012; 7: e36291 doi: 10.1371/journal.pone.0036291 22570700 10.1371/journal.pone.0036291 PMC3343050

[22] Noordhoek GT, van Embden JD, Kolk AH. Reliability of nucleic acid amplification for detection of *Mycobacterium tuberculosis*: an international collaborative quality control study among 30 laboratories. J Clin Microbiol. 1996; 34: 2522–2525. 8880513 10.1128/jcm.34.10.2522-2525.1996 PMC229309

[23] Muyldermans G, Soetens O, Antoine M, Bruisten S, Vincart B, Doucet-Populaire F, et al External quality assessment for molecular detection of *Bordetella pertussis* in European laboratories. J Clin Microbiol. 2005; 43: 30–35. doi: 10.1128/JCM.43.1.30-35.2005 15634947 10.1128/JCM.43.1.30-35.2005 PMC540137

[24] Pelloux H, Guy E, Angelici MC, Aspock H, Bessieres MH, Blatz R, et al A second European collaborative study on polymerase chain reaction for *Toxoplasma gondii*, involving 15 teams. FEMS Microbiol Lett. 1998; 165: 231–237. 9742693 10.1111/j.1574-6968.1998.tb13151.x

[25] Cruz I, Millet A, Carrillo E, Chenik M, Salotra P, Verma S, et al An approach for interlaboratory comparison of conventional and real-time PCR assays for diagnosis of human leishmaniasis. Exp Parasitol. 2013; 134: 281–289. doi: 10.1016/j.exppara.2013.03.026 23562705 10.1016/j.exppara.2013.03.026

[26] Murphy SC, Hermsen CC, Douglas AD, Edwards NJ, Petersen I, Fahle GA, et al External quality assurance of malaria nucleic acid testing for clinical trials and eradication surveillance. PLoS One. 2014; 9: e97398 doi: 10.1371/journal.pone.0097398 24838112 10.1371/journal.pone.0097398 PMC4023973

[27] Schijman AG, Bisio M, Orellana L, Sued M, Duffy T, Mejia-Jaramillo AM, et al International Study to Evaluate PCR Methods for Detection of *Trypanosoma cruzi* DNA in Blood Samples from Chagas Disease Patients. PLoS Negl Trop Dis. 2011; 5: e931 doi: 10.1371/journal.pntd.0000931 21264349 10.1371/journal.pntd.0000931 PMC3019106

[28] Piron M, Fisa R, Casamitjana N, Lopez-Chejade P, Puig L, Verges M, et al Development of a real-time PCR assay for *Trypanosoma cruzi* detection in blood samples. Acta Trop. 2007; 103: 195–200. doi: 10.1016/j.actatropica.2007.05.019 17662227 10.1016/j.actatropica.2007.05.019

[29] Burd EM. Validation of laboratory-developed molecular assays for infectious diseases. Clin Microbiol Rev. 2010; 23: 550–576. doi: 10.1128/CMR.00074-09 20610823 10.1128/CMR.00074-09 PMC2901657

[30] Duffy T, Bisio M, Altcheh J, Burgos JM, Diez M, Levin MJ, et al Accurate real-time PCR strategy for monitoring bloodstream parasitic loads in Chagas disease patients. PLoS Negl Trop Dis. 2009; 3: e419 doi: 10.1371/journal.pntd.0000419 19381287 10.1371/journal.pntd.0000419 PMC2667272

[31] Langton SD, Chevennement R, Nagelkerke N, Lombard B. Analysing collaborative trials for qualitative microbiological methods: accordance and concordance. Int J Food Microbiol. 2002; 79: 175–181. 12371652 10.1016/s0168-1605(02)00107-1

[32] Landis JR, Koch GG. The measurement of observer agreement for categorical data. Biometrics. 1977; 33: 159–174. 843571

[33] Burns MJ, Nixon GJ, Foy CA, Harris N. Standardisation of data from real-time quantitative PCR methods—evaluation of outliers and comparison of calibration curves. BMC Biotechnol. 2005; 5: 31 doi: 10.1186/1472-6750-5-31 16336641 10.1186/1472-6750-5-31 PMC1326201

[34] Ezzelle J, Rodriguez-Chavez IR, Darden JM, Stirewalt M, Kunwar N, Hitchcock R, et al Guidelines on good clinical laboratory practice: bridging operations between research and clinical research laboratories. J Pharm Biomed Anal. 2008; 46: 18–29. doi: 10.1016/j.jpba.2007.10.010 18037599 10.1016/j.jpba.2007.10.010 PMC2213906

[35] WHO/CDC/CLSI. Laboratory Quality Management System Training Toolkit. Module 10—Assessment—External Quality Assessment. In: Current Laboratory Practice Series [Internet]. 2009 [cited 9 Sep 2017]. Available: http://www.who.int/ihr/training/laboratory_quality/eqa_assessment/en/

[36] Martinez-Perez A, Poveda C, Ramirez JD, Norman F, Girones N, Guhl F, et al Prevalence of *Trypanosoma cruzi*’s Discrete Typing Units in a cohort of Latin American migrants in Spain. Acta Trop. 2016; 157: 145–150. doi: 10.1016/j.actatropica.2016.01.032 26851167 10.1016/j.actatropica.2016.01.032

